# Halofuginone Sensitizes Lung Cancer Organoids to Cisplatin *via* Suppressing PI3K/AKT and MAPK Signaling Pathways

**DOI:** 10.3389/fcell.2021.773048

**Published:** 2021-11-24

**Authors:** Hefei Li, Yushan Zhang, Xiaomei Lan, Jianhua Yu, Changshuang Yang, Zhijian Sun, Ping Kang, Yi Han, Daping Yu

**Affiliations:** ^1^ Department of Thoracic Surgery, Affiliated Hospital of Hebei University, Baoding, China; ^2^ Department of Thoracic Surgery, Beijing Chest Hospital, Beijing Tuberculosis and Thoracic Tumor Research Institute, Capital Medical University, Beijing, China; ^3^ K2 Oncology Co. Ltd., Beijing, China; ^4^ Oncology Department, Wang Jing Hospital of China Academy of Chinese Medical Sciences, Beijing, China

**Keywords:** lung cancer, patient-derived organoid, halofuginone, PI3K/AKT, MAPK

## Abstract

Lung cancer is the leading cause of cancer death worldwide. Cisplatin is the major DNA-damaging anticancer drug that cross-links the DNA in cancer cells, but many patients inevitably develop resistance with treatment. Identification of a cisplatin sensitizer might postpone or even reverse the development of cisplatin resistance. Halofuginone (HF), a natural small molecule isolated from *Dichroa febrifuga*, has been found to play an antitumor role. In this study, we found that HF inhibited the proliferation, induced G0/G1 phase arrest, and promoted apoptosis in lung cancer cells in a dose-dependent manner. To explore the underlying mechanism of this antitumor effect of halofuginone, we performed RNA sequencing to profile transcriptomes of NSCLC cells treated with or without halofuginone. Gene expression profiling and KEGG analysis indicated that PI3K/AKT and MAPK signaling pathways were top-ranked pathways affected by halofuginone. Moreover, combination of cisplatin and HF revealed that HF could sensitize the cisplatin-resistant patient-derived lung cancer organoids and lung cancer cells to cisplatin treatment. Taken together, this study identified HF as a cisplatin sensitizer and a dual pathway inhibitor, which might provide a new strategy to improve prognosis of patients with cisplatin-resistant lung cancer.

## Introduction

Lung cancer is the most commonly diagnosed cancer (11.4% of the total cases) and the leading cause of cancer death (18% of the total cancer deaths) worldwide (Sung et al., 2021). Although obvious progression has been made in surgical and pharmacological therapies for lung cancer, relapses of lung cancer are frequently documented with stronger drug resistance than primary tumor (Chen H.-Z. et al., 2021). Up to now, platinum and its derivatives are still the major choice for chemotherapy against cancers. However, platinum-based chemotherapy drugs are often confronted with the problem of drug resistance, and the cancers with drug resistance are usually incurable. Therefore, finding novel sensitizers which can be used in combination with platinum to improve the clinical utility of platinum has attracted close attention from researchers in oncology, pharmacology, and chemistry worldwide.

Increasing evidence suggests that the development of platinum resistance requires orchestration of multiple signaling pathways, including the PI3K/AKT and MAPK pathway (Fang et al., 2017; Liang et al., 2019). Inhibition of key pathways responsible for platinum resistance with a single multi-pathway inhibitor might result in better efficacy with low toxicity than combination of inhibitors targeting individual pathway (Chen et al., 2016; Sanchez et al., 2019). Therefore, phenotypic screening of large chemical libraries in clinically relevant disease models could be used to fulfill this purpose. The establishment of a 3D preclinical model, which could well recapitulate the derived primary tumor and portray the *in vivo* response more accurately, is urgently needed to screen potential natural molecules to resolve the issue of cisplatin resistance. Patient-derived organoids (PDOs) are novel preclinical models and closely resemble their primary tumors in both historical features and molecular characteristics, which have attracted more and more attention in both high-throughput drug screening, personalized drug design, and companion diagnostics for patients (Puca et al., 2018; Ooft et al., 2019; Pasch et al., 2019; Shi et al., 2019; Driehuis et al., 2020; Yao et al., 2020).

Halofuginone (HF) is a febrifugine-derivative alkaloid extracted from *Dichroa febrifuga*. It has been reported that HF possesses marked antimalarial (Hewitt et al., 2017), anti-coccidial (Matus and Boison, 2016), and anticancer activities (Akhtar et al., 2018; Xia et al., 2018). The anticancer properties of HF might be attributed to promote infiltration of favorable immune cells (Huang and Brekken, 2019), suppressing pathways on Smad3/TGF-β (Cui et al., 2016), AKT/mTOR, and/or p53 signaling (Akhtar et al., 2018; Xia et al., 2018), preventing the differentiation of fibroblasts to myofibroblasts and the transition of epithelial cells to mesenchymal cells in mammals, inhibiting prolyl-tRNA synthetase, activating the amino acid starvation response, preventing the differentiation of TH17 cells to blunt autoimmune responses, and triggering the autophagy (Chen et al., 2017; Xia et al., 2018). However, the evidence on the response of PDOs to HF in modeling the *in vivo* drug response remains missing.

In this study, we evaluated the effects of HF on cisplatin-resistant lung cancer PDOs and cells to determine whether it acted as a sensitizer. RNA sequencing of NCI-H1299 and NCI-H460, two lung cancer cell lines, treated with HF exhibited significant transcriptional alterations of genes involved in PI3K/AKT and MAPK signaling pathways. *In vitro* functional assays showed strong growth inhibition of HF. Thus, we proposed a hypothesis that HF exhibited anticancer properties in lung cancer cell lines by the dual regulation of MAPK and PI3K/AKT signaling pathways. Of note, HF exhibited a synergy effect with cisplatin in our studied cell lines. Taken together, our investigation illustrated that HF is a promising anticancer hit and sensitizer for cisplatin in lung cancer.

## Materials and Methods

### Tissue Processing and Organoid Culture

Lung cancer organoids were derived from surgery samples or transbronchial biopsies of lung cancer patients at Beijing Chest Hospital, Capital Medical University, Beijing, China. The study has got the approval of the Ethical Committee of Beijing Chest Hospital, Capital Medical University (Trial No. 11, 2020). Patients participating in this study signed informed consent forms. On arrival, tumor tissues were washed with cold PBS, cut into small pieces, washed with Advanced DMEM/F12 (Thermo Fisher Scientific, Waltham, MA, United States; containing 1× Glutamax, 10 mM HEPES and antibiotics), and digested with collagenase (Sigma-Aldrich, Cat #C9407, 2 mg/ml) for 1–2 h at 37°C. After washing twice with fresh medium (2% fetal calf serum, FCS) and centrifugation (400 rcf, 4 min), dissociated cells were seeded into growth factor reduced Matrigel (Corning, Cat # 356252) with the presence of Advanced DMEM/F12 at 37°C for 30 min. Next, the surface of the solidified mixture of cell suspension/Matrigel was sealed with complete human organoid medium (HOM, 500 μL), which comprised Advanced DMEM/F12 supplementing with series additives as described by Lampis et al. (2017) and Puca et al. (2018), replacing every 3 days. When the organoids ranged up to 200–500 μm in diameter (about 1 week), organoids were dissociated and passaged weekly using TrypLE Express (Gibco, Grand Island, NY, United States). The PDOs (2 × 10^6^ cells/tube, P3) were frozen using the Recovery Cell Culture Freezing Medium (Gibco) and stored at −80°C before drug screening.

### Compound Screening

A collection of almost 1,100 natural products were obtained from MedChemExpress (Shanghai, China). The natural product library was reformatted into 96-well source plates with concentration of 3.3 mM for automated robotic screening. At parallel, the cells were also treated with an equal volume (0.1%) of DMSO as a negative control and 1 μM final of staurosporine (MCE, shanghai, China) as a positive control. Plate-to-plate normalization and assay quality control were calculated according to them. A 3D cell viability assay was implemented, which determines the number of cell viability according to the ATP level using commercially available luminescence detection reagent (CellTiter-Glo 3D, #G9683, Promega, Madison, WI). In brief, organoids were processed as described earlier and plated in a 96-well low binding assay plate at a density of 6,000 cells per well in 50 μL comprising 10% growth factor reduced Matrigel. Additional 40 μL culture medium without Matrigel was added. Organoids were maintained in medium described earlier and drugged 2 days later by adding 10 μL culture medium comprising 33 μM natural product to get a final concentration of 3.3 μM (Supplementary Figure S1A). The assay was terminated at day 5 by adding 50 μL CellTiter-Glo. Assay quality and robustness were evaluated with the signal window (SW) and Z factor. Triplicate wells treated with staurosporine and vehicle solution (DMSO) were employed as bottom wells and top wells, respectively (Supplementary Figure S1B). The assay showed the signal windows (SW) were much larger than 10, and the Z factor values were between 0.5 and 1, which indicated that the assay was qualified for high-throughput screening (Supplementary Figures S1C,D).

### Cell Lines and Cell Culture

Human lung cancer cell lines NCI-H1299 (ATCC Cat# CRL-5803, RRID: CVCL_0060) and NCI-H460 (ATCC Cat# HTB-177, RRID: CVCL_0459) were purchased from the American Type Culture Collection (ATCC; Manassas, VA, United States). Cells were maintained in RPMI-1640/1641/1642 medium (Gibco) supplemented with 10% FCS (Gibco) and 1% penicillin–streptavidin (Gibco) at 37°C in 5% CO_2_.

### Cell Viability Assay and Foci Assay

NCI-H460 (0.75 × 10^3^ cells/well) and NCI-H1299 (0.75 × 10^3^ cells/well) cell lines were seeded into 96-well plates and treated with vehicle or HF for 1, 3, and 5 days. After incubation, cell viability was detected using luminescence detection reagent (CellTiter-Glo, #G9243, Promega, Madison, WI). The CellTiter-Glo assay determines the number of viable cells in culture by quantifying ATP, which indicates the presence of metabolically active cells. Luminescence readout is directly proportional to the number of viable cells in culture. As for the foci assay, NCI-H460 (2 × 10^3^ cells/well) and NCI-H1299 (2 × 10^3^ cells/well) were seeded into 6-well plates, treated with HF as the way of cell viability assay, then fixed and stained the cells with crystal violet solution, and took photos with a camera and bright-field microscope. Each vial of frozen cells was thawed and maintained for a maximum of 10 passages.

### Analysis of Cell Cycle Arrest and Apoptosis

The cell cycle and apoptosis were detected as previously described (Shi et al., 2018). Cells were cultured and treated with DMSO and HF (0.05 and 0.2 μM) in both NCI-H460 and NCI-H1299 for 24 h, followed by single staining with PI (Beyotime) for cell cycle analysis and double staining with PI and Annexin V-FITC (Beyotime) for apoptosis analysis. Data analysis was performed using NovoExpress v1.3.4.

### Western Blot Analysis

A standard Western blot analysis of whole-cell protein lysates was performed using primary antibodies against cleaved PARP, PARP (Cell Signaling Technology, #9542, 1:1,000, RRID:AB_2160739), cleaved caspase-3 (Cell Signaling Technology Cat# 9661, 1:1,000, RRID:AB_2341188), caspase-3 (Cell Signaling Technology Cat# 9662, 1:1,000, RRID:AB_331439), cylclinD1 (Cell Signaling Technology Cat# 2978, 1:1,000, RRID:AB_2259616), p27 (Cell Signaling Technology Cat# 3686, 1:1,000, RRID:AB_2077850), p21 (Cell Signaling Technology Cat# 2947, 1:1,000, RRID:AB_823586), pRb (Cell Signaling Technology Cat# 8516, 1:1,000, RRID:AB_11178658), and Rb (Cell Signaling Technology Cat# 9309, 1:1,000, RRID:AB_823629) to check the changes of apoptosis and G0/G1 phase markers. As well, for the alteration of effectors, the signaling pathways were examined with the primary antibodies including p-AKT1/2 (Cell Signaling Technology Cat# 4060, 1:1,000, RRID:AB_2315049), AKT1/2 (Proteintech Cat# 10176-2-AP, 1:2000, RRID:AB_2224574), p-ERK (Cell Signaling Technology Cat# 4370, 1:1,000, RRID:AB_2315112), and ERK (Proteintech Cat# 16443-1-AP, 1:2000, RRID:AB_10603369). Equal amounts of protein, which were blotted with an anti-β-actin antibody (Proteintech Cat# 60008-1-Ig, 1:2000, RRID: AB _2289225), was used as loading control.

### RNA-Seq Analysis

NCI-H460 and NCI-H1299 cells were incubated with DMSO or HF (0.05 and 0.2 μM) for 48 h; after harvest, total RNA was isolated using the TriZolTM UP Plus RNA Kit. RNA was sent to BGI (Beijing, China) for sequencing and analysis. In brief, after total RNA was fragmented into short fragments, mRNA was enriched using oligo (dT) magnetic beads, followed by cDNA synthesis. Double-stranded cDNA was purified and enriched by PCR amplification, after which the library products were sequenced using BGIseq-500. The heatmap of DEGs ( $\log_{2}FC$ ≥ 1, *p* ≤ 0.001) and KEGG analysis ( $\log_{2}FC$ ≥ 1, *p* ≤ 0.05) in NSCLC cell lines were performed by the BGI using the Dr. TOM approach, a customized data mining system from BGI. Altered (upregulated or downregulated) expression of genes was expressed as $\log_{2}FC$ , which represents log-transformed fold change ( $\log_{2}FC=\log_{2}\left[B\right]-\log_{2}\left[A\right]$ , while A and B represent values of gene expression for different treatment conditions).

### Dual Drug Combination Assay

NCI-H1299 and NCI-H460 cells were plated in 96-well plates and treated with various concentrations of cisplatin or/and HF, either alone or in combination, for 72 h. Cell viability was determined as described before. The synergy effect was evaluated by measuring the IC_50_ and highest single agent (HSA) reference model (Yadav et al., 2015). In addition, Western blotting analysis and flow cytometry assay were performed to detect the effect of drug combination on signaling pathways and cell cycle arrest.

Cisplatin-resistant PDOs (PDO-R1 and PDO-R2) were derived from two cisplatin-resistant patients and were plated in 96-well plates and treated with various concentrations of HF and cisplatin. Synergistic effects were observed under a microscope, and Western blotting assay was performed to detect the effect of drug combination on signaling pathways.

### Statistical Analysis

Data statistical analysis was performed using GraphPad Prism 8.0. The HF IC_50_ values were analyzed using non-linear regression (curve fit). The cell cycle and apoptosis data were analyzed using Excel using Student’s t test. A *p* value < 0.05 was considered as statistically significant. All data subjected to statistical evaluations were gathered with at least three independent repeats of experiments.

## Results

### Compound Screening and the Characterization of Cisplatin-Resistant NSCLC PDOs

**FIGURE 1:**
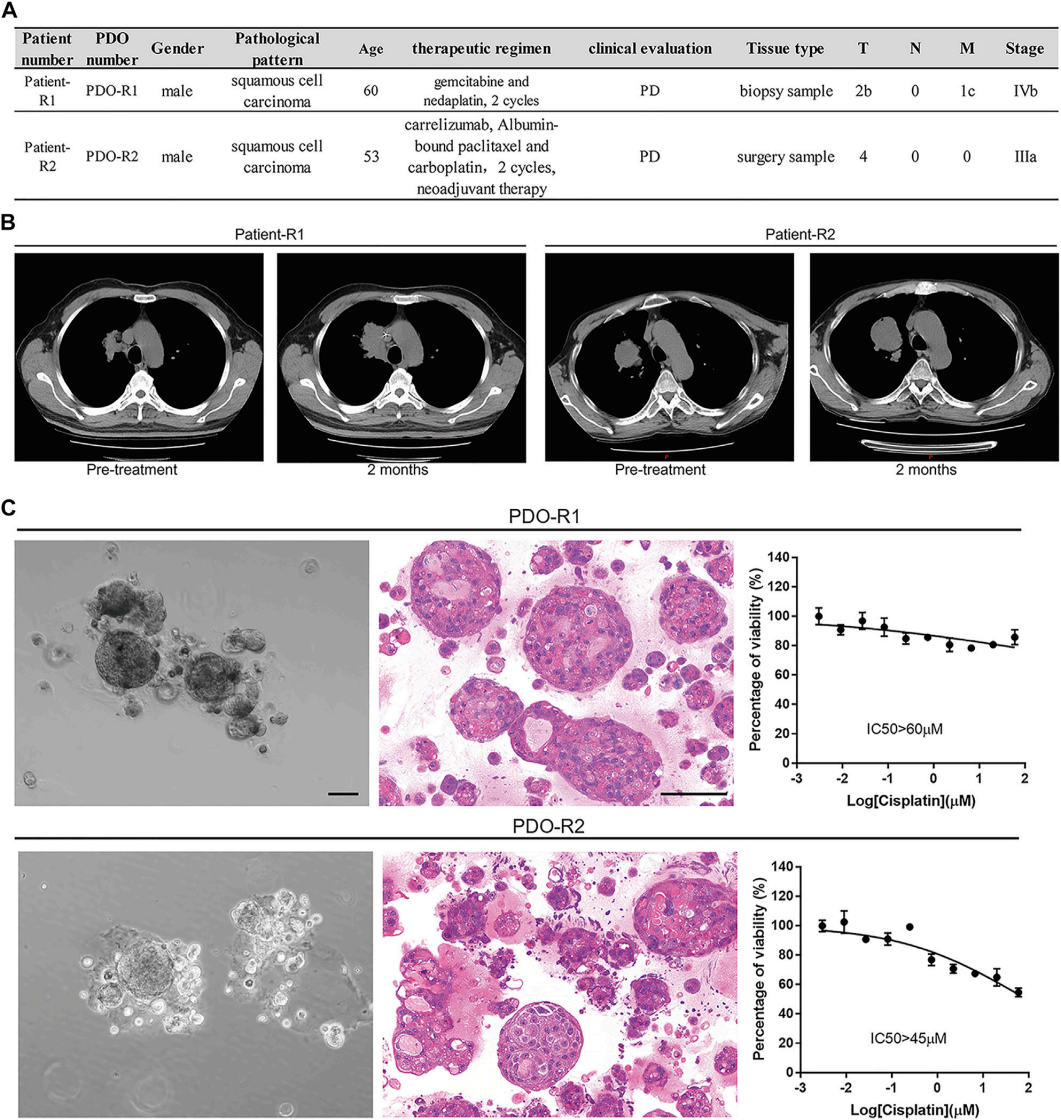
Patient-derived lung cancer organoids were generated and phenotypically represent the tumors from which they were derived. **(A)** Table of patients, diagnosis, clinical evaluation, and treatment status at time of surgery/biopsy where parent tumor was obtained. **(B)** Evaluation of patients’ clinical outcomes (computed tomography imaging of the subjects’ lung cancer tumors before and 2 months after treatment with platinum). **(C)** Bright-field images, H and E–stained images, and cisplatin-resistant characterization of PDO-R1 and PDO-R2.

PDOs have been reported for applications in preclinical drug discovery (Raja et al., 2016; Huang et al., 2017; Choudhury et al., 2020; Maloney et al., 2020; Skardal et al., 2020). A Two-stage screening strategy was employed whereby almost 1,100 natural products were primarily screened in 1 PDO and then validated in six PDO models which completed by K2 Oncology Co., Ltd. (Beijing 100061, P.R. China). The screening process is illustrated in Supplementary Figures S1A–D, and the results showed that HF is exactly one of the top hits with strong anticancer potential (Supplementary Figure S1E). Then we performed further anticancer effect verification of HF in two cisplatin-resistant lung cancer PDOs. The clinical information and data of these two PDOs are summarized in Figure 1A. Both patients were treated with the platinum-based regimen, and the RECIST evaluation was performed after two cycles (Figure 1B). The cisplatin resistance were maintained after PDOs were successfully established (Figures 1C,D).

### HF Inhibited Cell Growth in Lung Cancer Cell Lines

**FIGURE 2:**
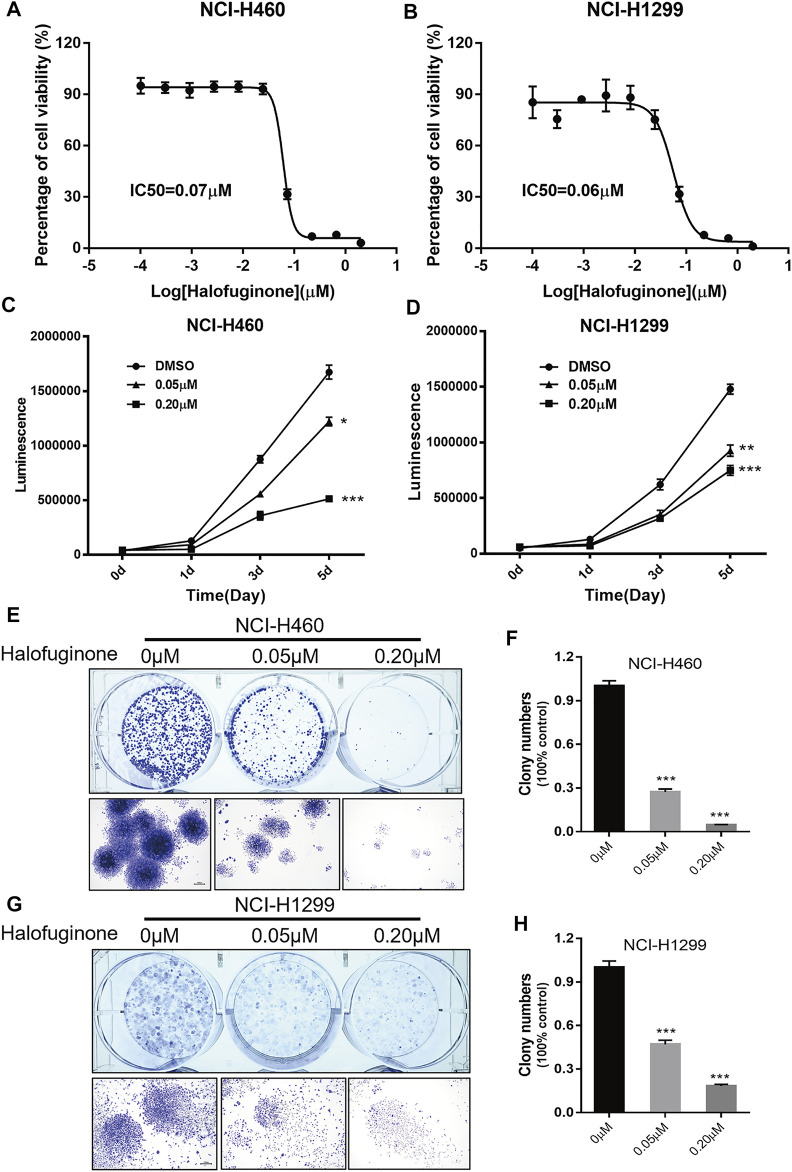
Halofuginone significantly inhibited cell growth in NCI-H460 and NCI-H1299 cells. **(A,B)** The dose–response curves and IC50 values of halofuginone in NCI-H460 and NCI-H1299, respectively. **(C,D)** Cell proliferation suppression in NCI-H460 and NCI-H1299 with the treatment of halofuginone at the concentration of 0.05 and 0.2 μM for 5 days. (**E–H)** Halofuginone inhibited the colony formation in NCI-H460 and NCI-H1299 cells at the concentration of 0.05 and 0.2 μM for 7 days. Cells were stained with crystal violet solution at the endpoint. (*p < 0.05, **p < 0.01, ***p < 0.001).

Next, we verified the effect of HF in lung cancer cell lines, NCI-H1299 and NCI-H460. NCI-H460 showed partial cisplatin sensitivity, but the maximal inhibition rate was very limited (Figure 4B). NCI-H1299 is a cisplatin-resistant lung cancer cell line (Fan et al., 2020) (Chen M. et al., 2021), and its cisplatin resistance is also validated in this study (Figure 4D). The dose–response curve of HF in both cell lines exhibited identical IC_50_ (0.07 μM) (Figures 2A,B). The significant dose-dependent and time-dependent inhibition of HF on lung cancer cell proliferation were observed in both cell lines (Figures 2C,D). In addition, HF remarkably suppressed colony formation of the two cell lines in a dose-dependent manner (Figures 2E–H). Thus, these results indicated a remarkable growth inhibition of HF in lung cancer cell lines.

### HF Induced G0/G1 Phase Arrest and Apoptosis in Lung Cancer Cell Lines

**FIGURE 3:**
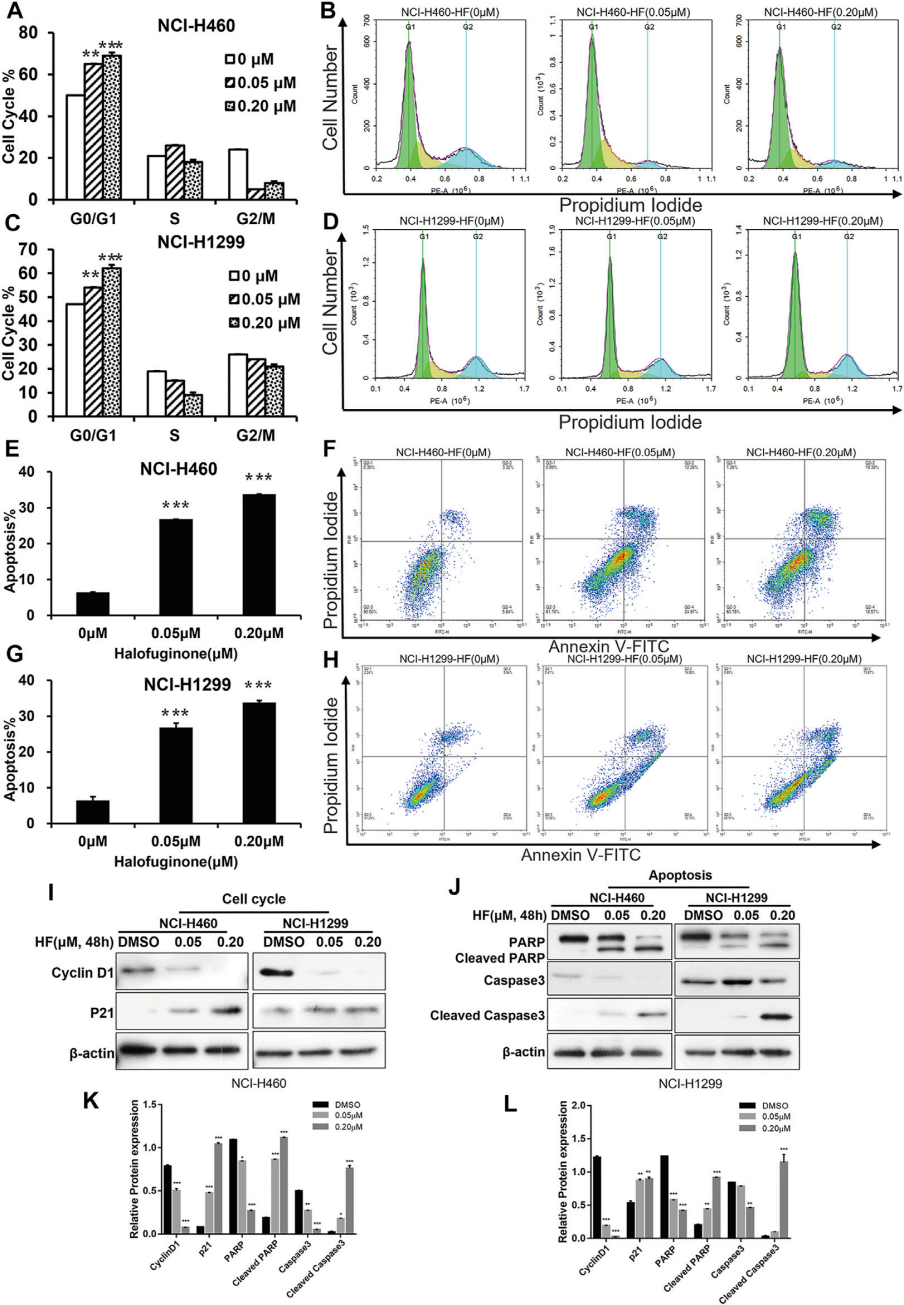
Halofuginone induced G0/G1 arrest and apoptosis in NCI-H460 and NCI-H1299 cells. **(A–D)** Flow cytometry analysis of the cell cycle by PI staining in NCI-H460 and NCI-H 1299 cells with treatment of DMSO or halofuginone for 48 h (*n* = 3). **(E–H)** Flow cytometry analysis of apoptosis analysis by PI/Annexin V-FITC staining in both NCI-H460 and NCI-H1299 cells with treatment of DMSO or halofuginone for 48 h (*n* = 3). **(I–L)** Cell cycle and apoptosis markers (cyclin D1, p21, PARP, cleaved PARP, and cleaved caspase 3) analysis by Western blot. β-actin was used as a loading control (*n* = 3) (*p < 0.05, **p < 0.01, ***p < 0.001).

To explore the reasons for growth inhibition of lung cancer cells by HF, cell cycle distribution, and apoptosis induction were evaluated on HF-treated lung cancer cell lines. Through flow-cytometry assay, we noticed a dose-dependent G0/G1 phase arrest (Figures 3A–D) in both NCI-H1299 and NCI-H460 following 24-h treatment of HF. Additionally, HF induced dose-dependent apoptosis in both cell lines (Figures 3E–H). In detail, both the early and late apoptosis were significantly induced by HF (Supplementary Figure S2). Cyclin D1 (Sherr, 1996; Lukas et al., 1996) and p21 (Cheng et al., 1999; Pestell et al., 1999) are two canonical cell cycle markers for the G1 phase. Western blotting confirmed G0/G1 phase arrest with the decreased cyclin D1 expression and increased p21 expression in accordance with the flow cytometry tendency (Figure 3I). PARP (Lu et al., 2019) and caspase (Julien and Wells, 2017) activation are the key events of apoptosis, and the expression levels of their full-length and cleaved forms were examined with Western blotting. The decreased PARP and caspase 3 and the increased cleaved PARP and cleaved caspase3 suggested that HF induced apoptosis in a dose-dependent manner as well (Figure 3J). Taken together, HF induced G0/G1 phase arrest and apoptosis in a dose-dependent manner in NCI-H460 and NCI-H1299, which well explained the significant lung cancer cells growth inhibition.

### HF Sensitized Lung Cancer Cell Lines to Cisplatin

**FIGURE 4:**
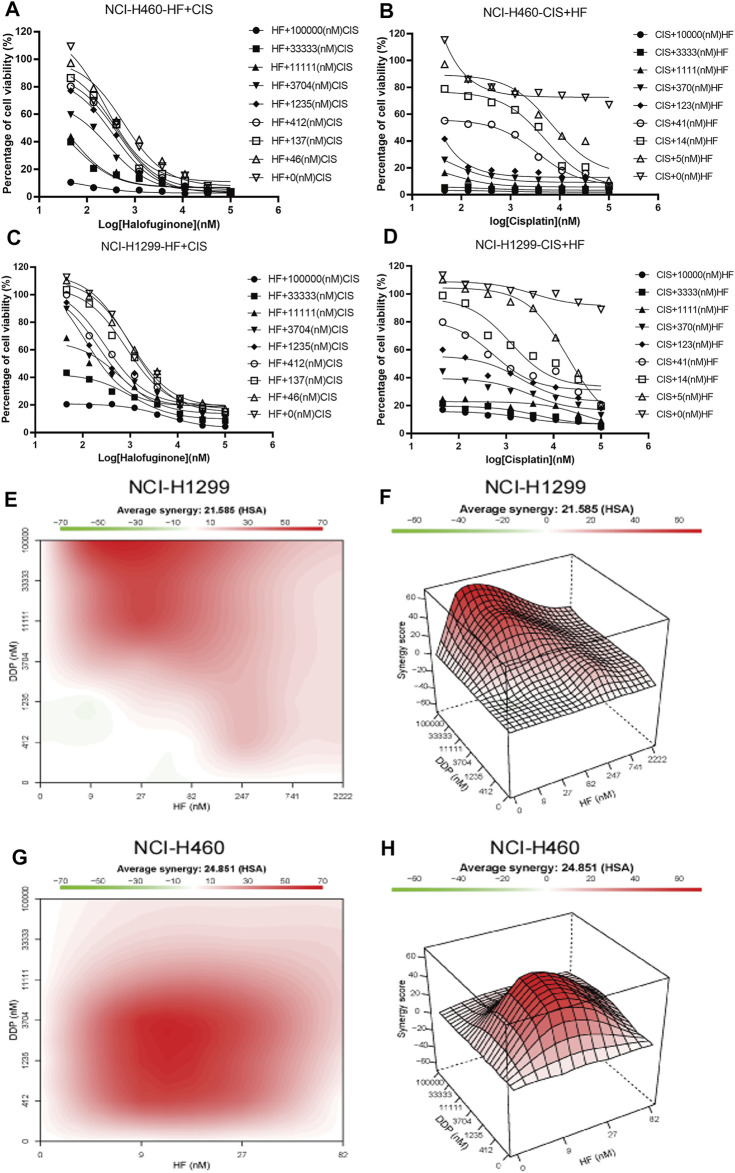
Synergy effects of halofuginone and cisplatin on cell viability of NCI-H460 and NCI-H1299. **(A–D)** The dose–response curves of NCI-H460 and NCI-H1299 cells treated with the combination of halofuginone and cisplatin. **(E–H)** The surface plot and heatmap show the Excess over the highest single agent (EOHSA) of halofuginone and cisplatin combination in NCI-H460 and NCI-H1299 cells.

To further investigate the impact of HF on cisplatin resistance in lung cancer, lung cancer cell lines, NCI-H1299 and NCI-H460, were employed for the dural drug synergy test. NCI-H1299 shows resistance to cisplatin, with IC_50_ larger than 100 mM and maximal inhibition rate smaller than 10%. NCI-H460 showed limited response to cisplatin, with maximal inhibition rate of about 20%, which induced the absolute IC_50_ larger than 10 mM and indicated a very low efficacy (Figures 4B,D). The synergy effect was evaluated by measuring the IC_50_ and the highest single agent (HSA) reference model (Yadav et al., 2015). With the existence of HF, significant left shift of dose–efficacy curve and decrease in IC_50_ were observed (Figures 4B,D). By adding 5, 14, and 41 nM of HF, the IC_50_ of cisplatin decreased from larger than 10 mM to 8, 2.4, and 0.4 μM for NIC-H460 and 9, 2.5, and 0.5 μM for NCI-H1299, respectively. Consistently, by adding serial diluted cisplatin to HF, the IC_50_ of HF also decreased, respectively (Figures 4A,C). To further analyze the synergy effect of cisplatin with HF, synergy score matrixes were calculated with the highest single agent (HSA) reference model. The average synergy score of cisplatin with HF is 21.585 and 24.851 for NCI-H1299 and NCI-H460, respectively. The HSA synergy scores were visualized with heatmap (red areas in the model graph) and 3D hillmap (Figures 4E–H). These data suggest the synergic effect of HF and cisplatin in lung cancer cell lines.

**FIGURE 5:**
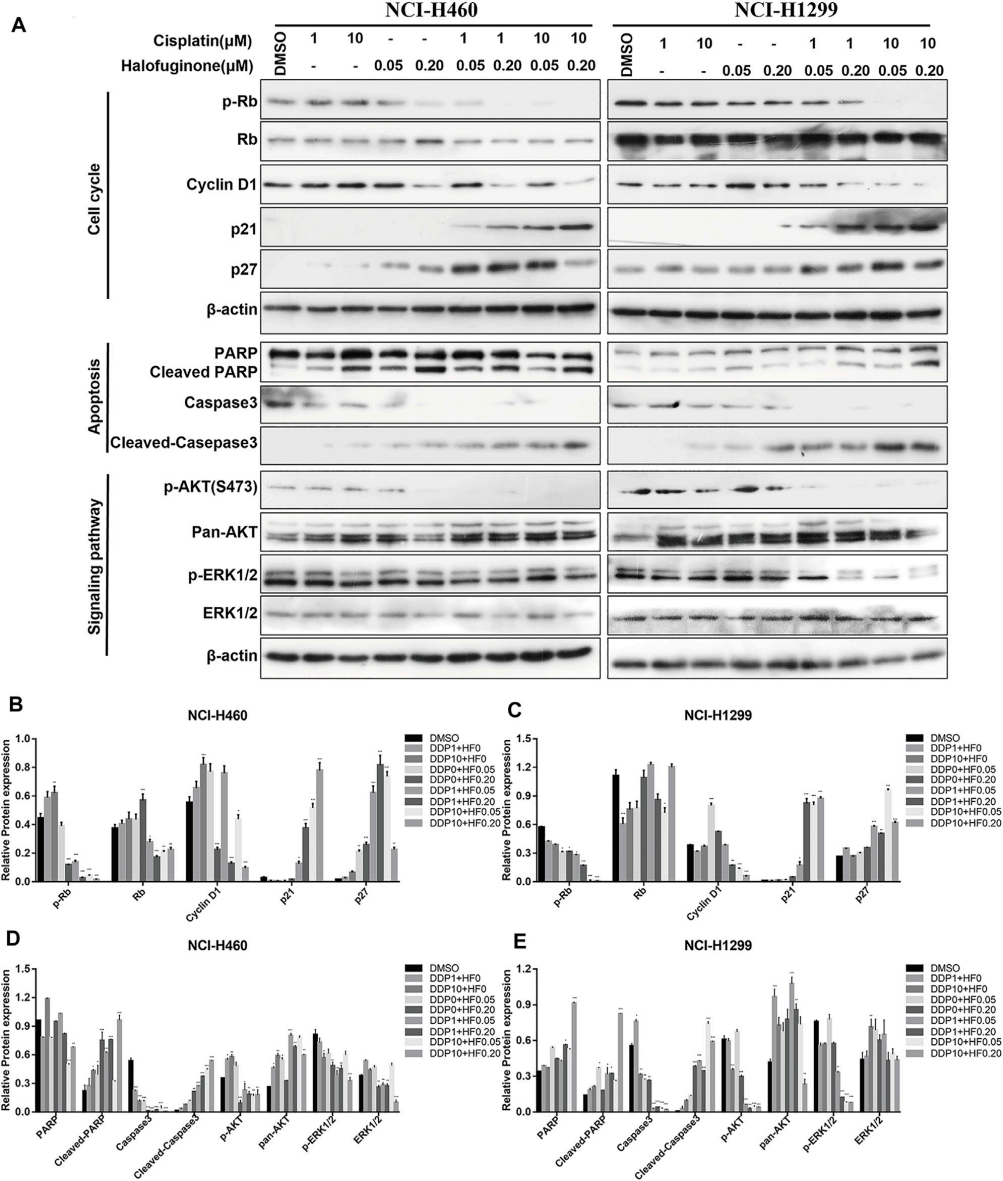
Expression of markers related to cell cycle, apoptosis, and proliferation was detected by Western blotting in NCI-H460 and NCI-H1299 cells treated with the combination of halofuginone and cisplatin. **(A)** The cell lysates from NCI-H460 and NCI-H1299 cells were analyzed by Western blotting using the indicated antibodies. **(B-E)** Relative protein expressions were quantified by Image J software. (*n* = 3, *p < 0.05, **p < 0.01, ***p < 0.001).

**FIGURE 6:**
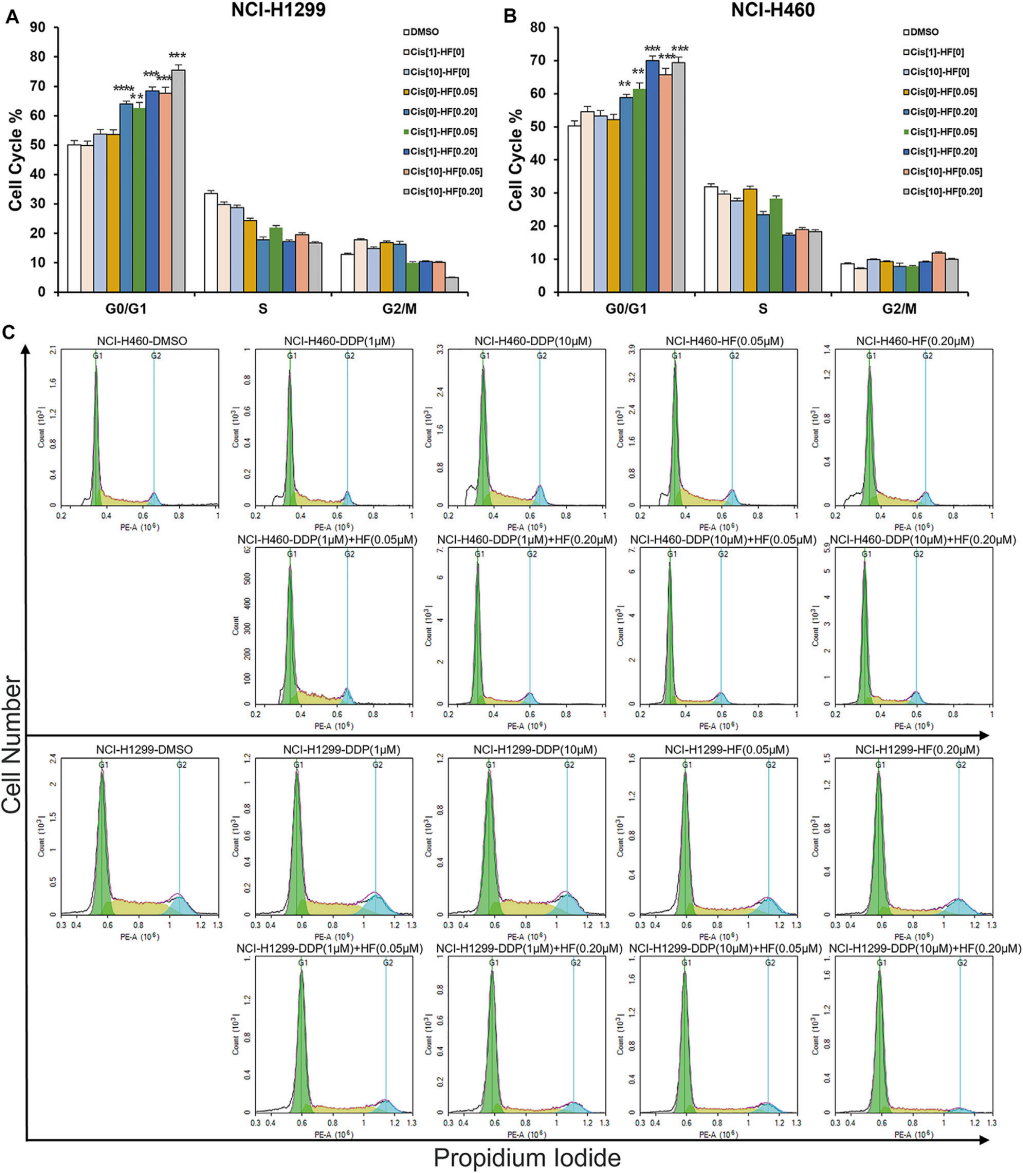
Halofuginone/cisplatin combination significantly induced G0/G1 arrest in NCI-H1299 and NCI-H460 cells (*n* = 3, *p < 0.05, **p < 0.01, ***p < 0.001).

To further explore the potential value and the underlying synergy mechanism of HF with cisplatin, 1 and 10 μM of cisplatin and 50 and 200 nM HF were selected for cell cycle, apoptosis, and pathway markers detection using Western blot in NCI-H460 and NCI-H1299. When HF was combined with 1 μM of cisplatin, significant G0/G1 phase arrest was observed with the increase in p21 and p27 and the decrease in p-Rb and cyclin D1 in a dose-dependent manner (Figure 5 upper). HF combined with 10 μM of cisplatin showed similar trend but a stronger dose-dependent synergistic effect than low-dose cisplatin. In addition, the apoptosis markers also showed similar synergy effect with the indicator of caspase 3 and cleaved caspase 3 (Figure 5 middle). Similar tendency of G0/G1 phase arrest was further approved using flow cytometry with cell models treated with cisplatin alone, HF alone, and cisplatin combined with HF (Figures 6A–C). Obviously, cisplatin alone barely changed the cell cycle progression, but HF could induce prominent G0/G1 phase arrest at 200 nM. Altogether, these data demonstrated that HF would be a potential cisplatin sensitizer *via* G0/G1 phase arrest and apoptosis.

### HF Altered Genome-Wide Gene Expression in Lung Cancer Cell Lines

**FIGURE 7:**
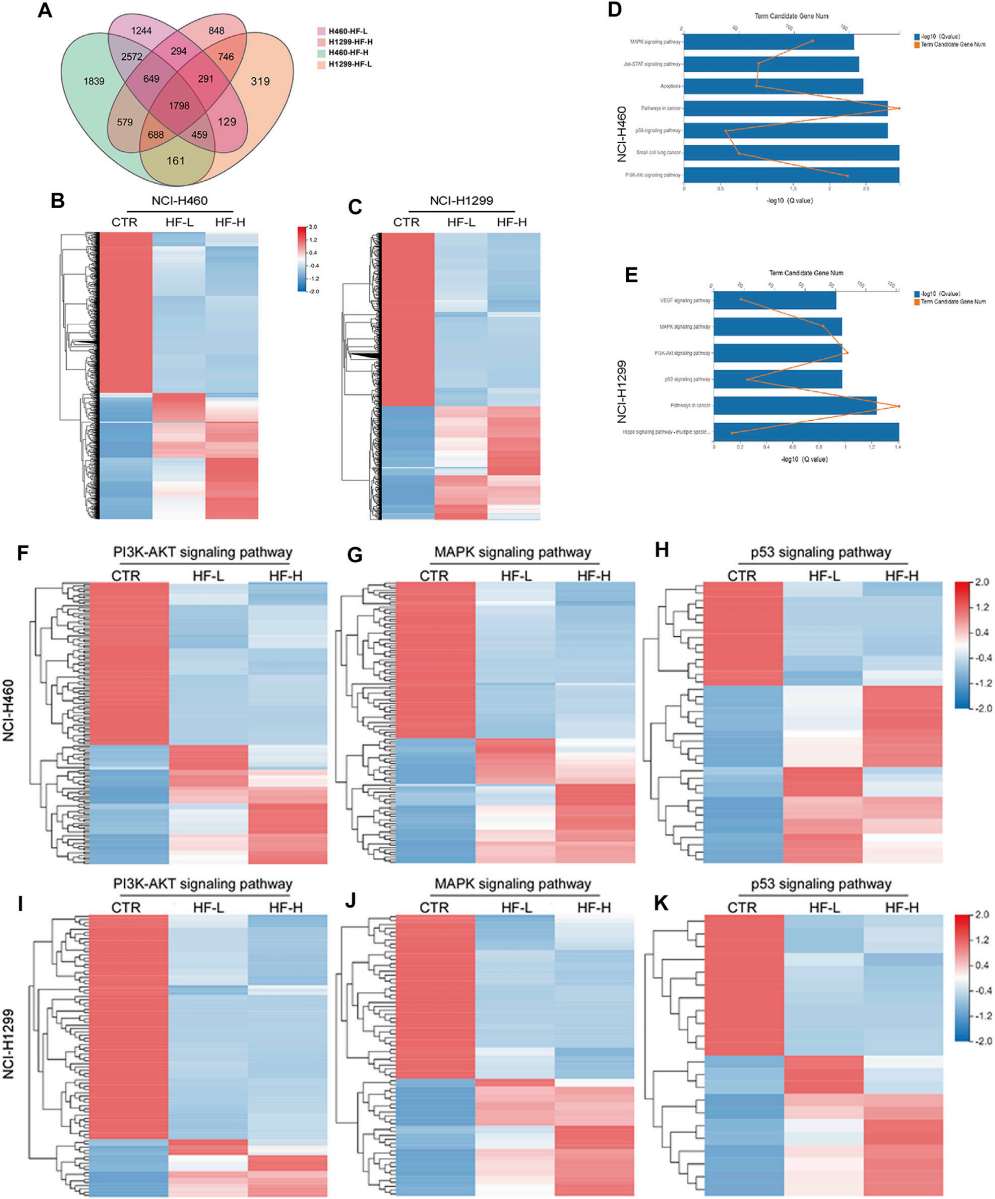
Halofuginone induced genome-wide gene alteration in NCI-H1299 and NCI-H460 cells (*n* = 3). **(A)** 1798 different expression genes (DEGs, log_2_FC ≥ 1, *p* ≤ 0.001) were identified both in NCI-H460 and NCI-H1299 with the treatment of different concentration of HF. **(B,C)** Heatmap of the differential expressed genes for NCI-H460 and NCI-H1299 cells. HF led to alterations of 5478 DEGs in NCI-H460 cells and 3523 DEGs in NCI-H1299 cells *versus* each corresponding control group. Red indicates high expression, and green indicates low expression. **(D,E)** KEGG analysis of the DEGs revealed enrichments of cancer-associated pathways, including PI3K/AKT and MAPK signaling pathways. **(F–J)** Heatmap of the differential expressed genes related with PI3K/AKT MAPK and p53 signaling pathways.

RNA sequencing was performed to profile gene expression in both NCI-H460 and NCI-H1299 with the treatment of HF at 0.05 and 0.2 μM, respectively. (The datasets presented in this study can be found in online repositories. The names of the repository and accession number can be found below: http://www.ncbi.nlm.nih.gov/sra, accession number PRJNA769938). In both cell lines, >4000 genes were differentially expressed under the two concentrations. We observed substantial overlap between the two cell lines with regard to the genes that were differentially expressed following incubation with HF-1798 genes (DEGs, log_2_FC ≥ 1, *p* ≤ 0.001) were shared in NCI-H460 and NCI-H1299 with the treatment of different concentration of HF (Figure 7A). In addition, HF led to alterations of 5478 DEGs in NCI-H460 cells and 3523 DEGs in NCI-H1299 cells *versus* each corresponding control group (Figures 7B,C). The KEGG analysis of the DEGs revealed enrichments of cancer-associated pathways, including PI3K/AKT and MAPK signaling pathways (Figures 7D,E). Moreover, the genes related with PI3K/AKT and MAPK signaling pathways were sensitive to HF exposure (Figures 7F,G,I,J).

### HF Blocked PI3K/AKT Signaling Pathway in Lung Cancer Cell Lines

Upregulation of p-AKT might enhance tumor progression and mediate resistance to drugs (Liu R. et al., 2020), and it has been well known that the binding of ligand to a transmembrane receptor, receptor tyrosine kinase (RTK), activates both PI3K/AKT and MAPK pathways to promote cell survival (Cao et al., 2019). Herein, we hypothesized that dual inhibition on the two pathways would exert a more stable and stronger growth and survival suppression than targeting individual pathway. The ratio of p-AKT (S473) to total Akt examined by Western blot was decreased by HF in a dose-dependent manner (Figure 5 lower). Here, the observed downregulation of p-Akt in our cisplatin-resistant lung cancer cell lines by HF, indicating possible tumor suppression. Together, HF inhibited the cisplatin-resistant cell models growth *via* blocking the PI3K/AKT signaling pathway.

### HF Blocked MAPK Signaling Pathway in Lung Cancer Cell Lines

The identical analysis and assays as before were performed to examine the effect of HF on the MAPK signaling pathway, which includes a small G protein (Ras) and three protein kinases (Raf, Mek, and Erk) and is activated with translocation of Erk (MAPK) to the nucleus (McCain, 2013). In protein level, p-Erk/Erk examined by Western blot exhibited dose-dependent decrease with the exposure of HF (Figure 5 lower). Activation of the MAPK signaling pathway would strengthen tumor progression and mediate drug resistance as well (Cocco et al., 2019; Li et al., 2019). The decline of p-Erk1/2 indicated the potential activity of HF in lung cancer suppression. Thus, HF inhibited the cell models growth simultaneously *via* blocking the MAPK signaling pathway.

### HF Sensitized Cisplatin in Cisplatin-Resistant PDOs

**FIGURE 8:**
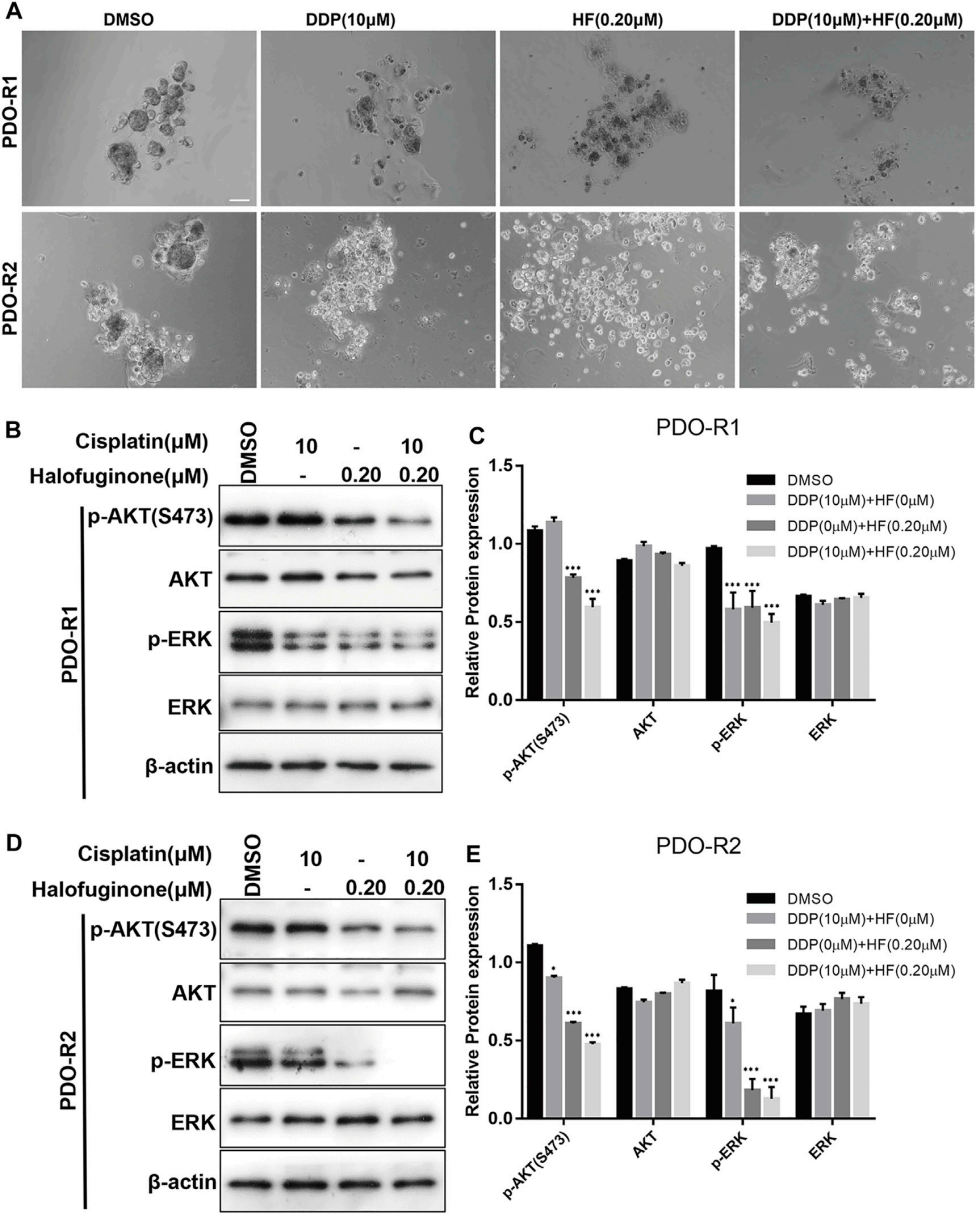
Synergy effects of halofuginone and cisplatin on cell viability of PDO. **(A)** Microphotograph images of cisplatin-resistant PDOs (PDO-R1 and PDO-R2) at indicated time points of treated by cisplatin alone, halofuginone alone, and cisplatin/halofuginone combination. **(B–E)** the expression levels of pAKT and p-ERK were detected by Western blotting in cisplatin-resistant PDOs treated with cisplatin, halofuginone alone, and halofuginone/cisplatin combination (*n* = 3) (*p < 0.05, **p < 0.01, ***p < 0.001).

To study the response to the combination treatment of cisplatin and HF, the efficiency of tumor destruction was observed under a microscope, and exposure of PDOs to HF and combination of cisplatin and HF led to substantially reduced survival (Figure 8A). In addition, significant p-AKT (S473) and p-ERK decrease *via* Western blot appeared in cisplatin and HF combination groups compared to single drug treatment groups or control group (Figures 8B–E). Therefore, the results suggested that HF had the capacity to expand its sensitizer effect for cisplatin to preclinical cisplatin-resistant PDO models.

## Discussion

Cisplatin is one of the most widely used chemotherapy agents in the treatment of lung tumors. The mechanism of action for cisplatin is considered as damaging DNA and inhibiting DNA synthesis. However, cancer cells would develop multi-type resistance to overcome DNA damage and synthesis suppression to diminish the therapeutics efficacy (Liao et al., 2020; Yang et al., 2020). Therefore, combination strategy with cisplatin and cisplatin sensitizer will be of promising clinical value. Accumulating evidence has indicated that activation of cell proliferation and survival pathways, such as PI3K/AKT and MAPK signaling pathways, contribute to cisplatin resistance (J, 2013). PDOs of cancers derived from cisplatin-resistant tumor tissue can be used to facilitate discovery of anticancer leading compounds for their close morphological and genetic features of the original tumor (Lancaster and Knoblich, 2014; Weeber et al., 2015; Boretto et al., 2019; Ooft et al., 2019; Ubink et al., 2019). Besides, the (pre)clinical efficiency and safety of multi-target natural products with potential capacity for multi-target drug discovery have been characterized by more and more studies (Koeberle and Werz, 2014; Chen et al., 2016; Gonçalves and Romeiro, 2019; Liu X. et al., 2020; Ren et al., 2020). Especially, for some complicated diseases, such as acute ischemic stroke (Chen et al., 2016), tissue plasminogen activator is the only FDA-approved drug for treatment, but its clinical use is limited by the narrow therapeutic time window and severe side effects. Adjunct therapies *via* a multi-target strategy are warranted in reducing the side effects and extending tissue plasminogen activator’s therapeutic time window with the consideration of the unsatisfaction of single target modulating. In addition, phenotype alteration induced by multi-target natural products could provide valuable information for drug combination (Sanchez et al., 2019).

Previous studies have reported that HF exhibited anticancer effects on various tumor types (Cui et al., 2016; Koohestani et al., 2016; Chen et al., 2017; Tsuchida et al., 2017; Akhtar et al., 2018; Xia et al., 2018; Huang and Brekken, 2019; Kunimi et al., 2019), which strongly supports the hypothesis that the therapeutic potential of HF is mainly through inducing anti-proliferation, autophagy, and apoptosis. However, the role and underlying mechanism of HF in cisplatin-resistant lung cancer cells have rarely been investigated. In this study, HF was found to be one of the top hits among 1,100 natural products in reducing cell viability of PDO models established by K2 Oncology Co., Ltd. (Beijing 100061, China).

Mechanically, we observed HF remarkably induced G0/G1 phase arrest and apoptosis in lung cancer cell lines. Subsequently, RNA sequencing was introduced for gene expression profiling regulated by HF. DEG analysis and KEGG analysis indicated that PI3K/AKT, MAPK, and p53 signaling pathways were affected. HF was once reported as a positive control drug that showed modest interaction of −6.91 kcal/mol having a Ki value of 8.61 μM with unbound p53 but expressed significant inhibition (Ki = 3.88 μM) against p21^Waf1/Cip1^ with binding energy of −7.38 kcal/mol. A multiplex analysis of phosphorylation of diverse components of signaling cascades revealed that HF induced changes in P38 MAPK activation and increased phosphorylation of c-Jun and p53. Our data exhibited significantly p27 and p21 increase, p-Rb and cyclin D1 decrease with the combination of cisplatin and HF. p27 and p21 are two downstream effectors of PI3K/AKT that led to G0/G1 phase arrest. Moreover, the p53 signaling pathway is a downstream part of the p38/MAPK signaling pathway which contributed to G0/G1 phase arrest. As the G0/G1 phase is the most sensitive period for tumor cells to cisplatin, disruption of the cell cycle at this period might be part of the reasons for cisplatin-resistant sensitization.

HF dose-dependently suppressed Akt and ERK signaling pathways, which indicated that HF suppressed the cisplatin-resistant cells by the dual targeting of PI3K/AKT and MAPK signaling pathways. Moreover, the combination study between cisplatin and HF qualitatively evaluated in lung cancer cell lines showed increased sensitivity to cisplatin with HF exposure, and the combination of cisplatin with both Akt inhibitor and ERK inhibitor also simulated this phenomenon. To further validate the preclinical synergy, two cisplatin-resistant PDO models were employed with the treatment of vehicle, HF alone, cisplatin alone, or HF combined with cisplatin. Additionally, the growth inhibition and dual signaling pathway suppression in the HF alone group and combination group in cisplatin-resistant PDOs were in line with that in lung cancer cell lines. Thus, HF might be a potential sensitizer to cisplatin by its dual pathway targeting effects.

Although this study showed that HF suppressed the cisplatin-resistant cells by the dual targeting of PI3K/Akt and MAPK signaling pathways, a broader insight into its multi-mechanistic nature requires a system-wide screening approach. As we all know, resistance to cisplatin is attributed to three molecular mechanisms: increased DNA repair, altered cellular accumulation, and increased drug inactivation. One of the most predominant mechanisms is the increase in DNA damage repair, among which nucleotide excision repair (NER) and mismatch repair (MMR) are included. Wang et al. indicated that HF significantly promoted DNA damage-related protein g-H2AX, pATM, and pATR expression in human esophageal cancer cell lines (Wang et al., 2020), informing HF is probably involved in the balance of DNA damage and DNA repair which decide cell death versus survival. So, further studies are required to examine the influence of HF on DNA damage repair.

P53 signaling is also associated with cisplatin resistance based on the literature evidence, and it was reported that HF could decrease the expression of p53 to suppress the migration and invasion in breast cancer cells (Xia et al., 2018). In this study, the RNAseq also indicted that the p53 pathway was regulated by HF treatment. The effect of HF on the p53 pathway will be further investigated in follow-up studies, which may provide a new vision of HF on p53 pathway drug discovery. There is still no specific p53 agonist in clinical development, which restricts the clinical translation of this study. By understanding the dural inhibitor effect to these pathways also provides evidence the clinical application of PI3Ki/AKTi and ERKi in cisplatin-resistant lung cancer.

Collectively, natural product has a huge multi-target library for synergy study and multi-target lead finding. By high-throughput clinical associated PDO models screening and RNA sequencing, we could get better understanding of multi-pathway networks and obtain more precious natural anticancer molecules. Altogether, this study demonstrated that HF might act as a promising therapeutic agent to sensitize cisplatin in the clinical chemotherapy strategy in cisplatin-resistant lung cancer and could be applied in clinical in future.

## Data Availability

The datasets presented in this study can be found in online repositories. The names of the repository/repositories and accession number(s) can be found NCBI with accession PRJNA769938.
