# Supplementary material for: Halofuginone Sensitizes Lung Cancer Organoids to Cisplatin via Suppressing PI3K/AKT and MAPK Signaling Pathways
Source: Front Cell Dev Biol. 2021 Nov 24;9:773048. doi: 10.3389/fcell.2021.773048 (PMC8652204; doi:10.3389/fcell.2021.773048)
Supplement: Supplementary file 1 [file DataSheet1.docx]

Supplementary Material

**
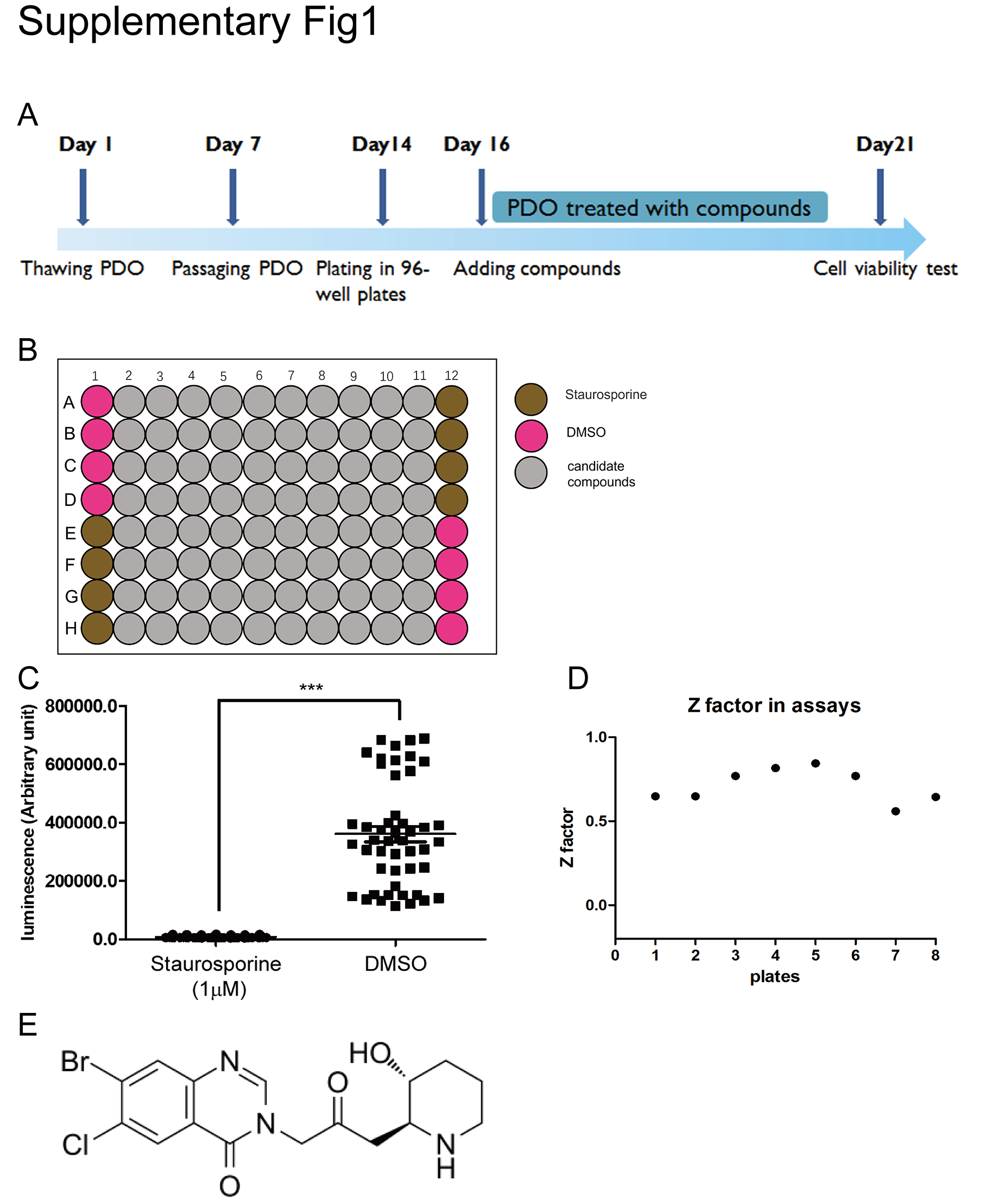
**

**Supplementary Figure 1.** Quality evaluation mentioned in the PDO drug screening and security parameter of HF. (**A**) the treatment scheme of drug screening using lung cancer PDOs. (**B**) plate design of high-throughput screening. **(C-D)** Assay quality and robustness were evaluated with signal window (SW) and Z factor. Triplicate wells treated with staurosporine and vehicle solvent (DMSO) were employed as bottom wells and top wells respectively. The assay showed the SW were much larger than 10 and the Z factor values were between 0.5 and 1, which indicate the assay was qualified for high-throughput screening. (**E**) the structure of halofuginone.


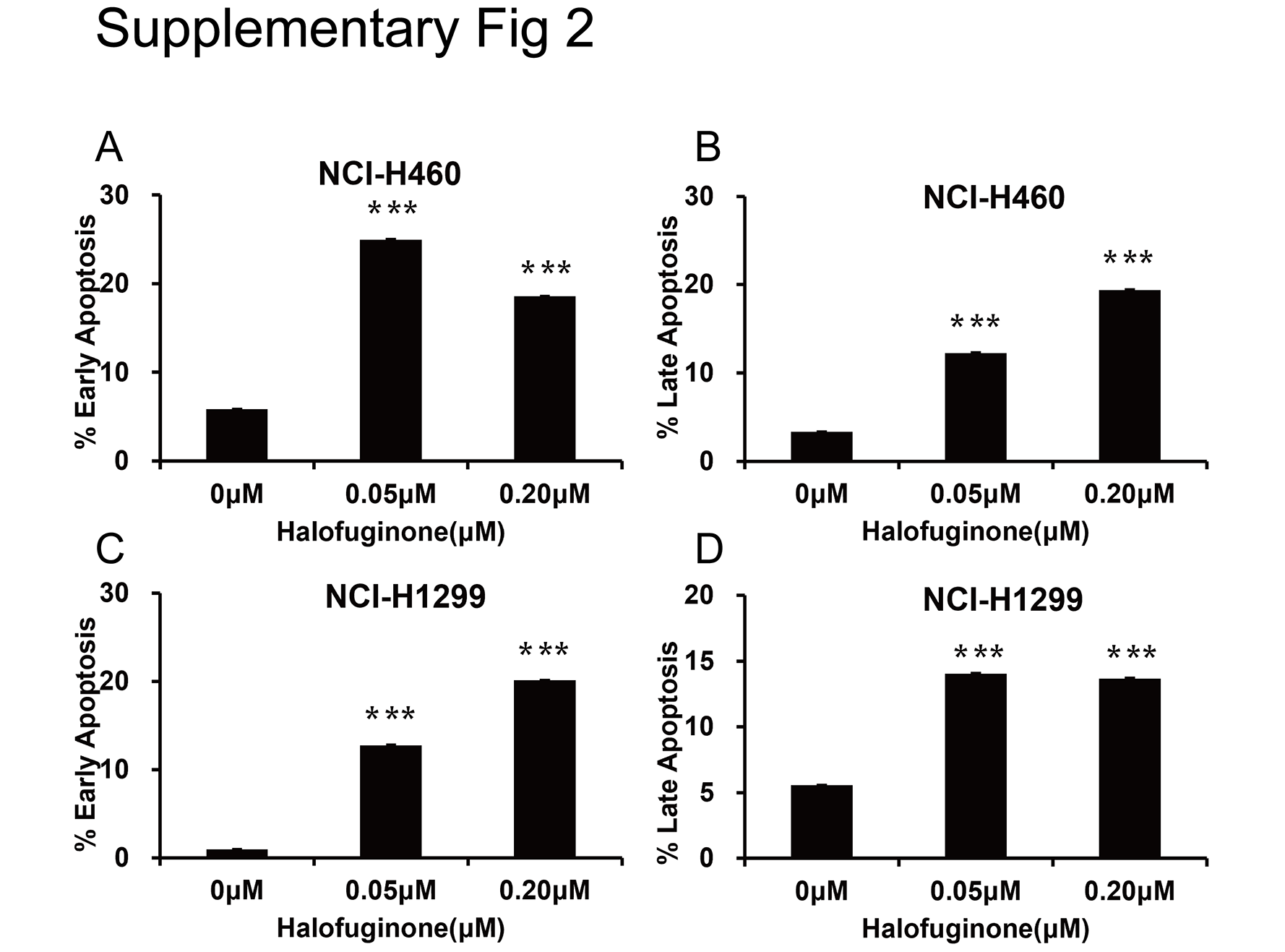


**Supplementary Figure 2.** HF induced both early apoptosis and late apoptosis in NCI-H460 and NCI-H1299 cells.

**
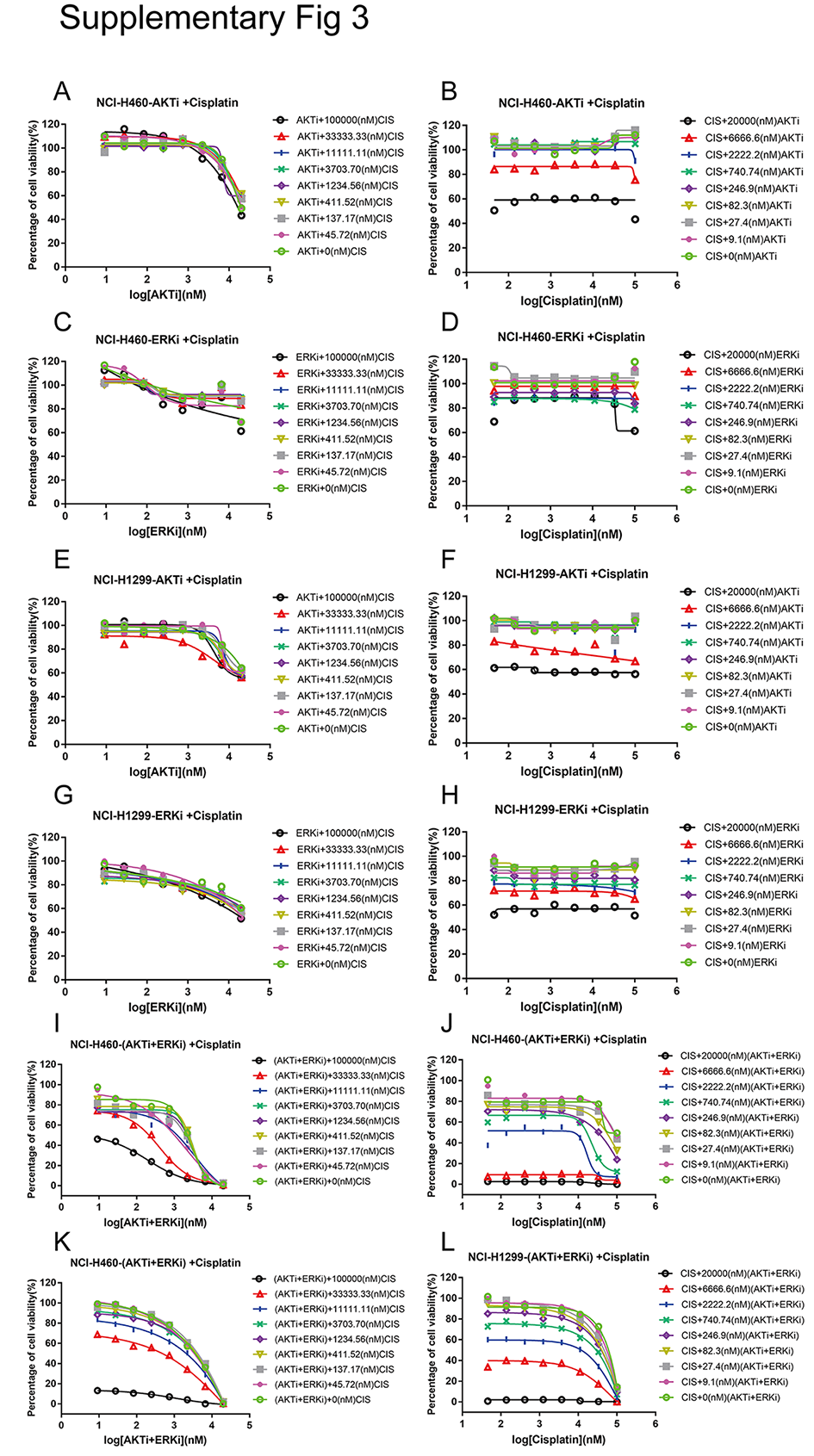
**

**Supplementary Figure 3.** The combination of cisplatin with AKT inhibitor ipatasertib and ERK inhibior ravoxertinib dose-dependently increased sensitivity to cisplatin in NCI-H460 and NCI-H1299 cells.
